# Development of superficial lung lesions monitored on farm by serial ultrasonographic examination in sheep with lesions confirmed as ovine pulmonary adenocarcinoma at necropsy

**DOI:** 10.1186/s13620-018-0134-0

**Published:** 2018-11-05

**Authors:** P. R. Scott, M. P. Dagleish, C. Cousens

**Affiliations:** 1 Capital Veterinary Services, West Latchfields, Scotland, EH41 4JN UK; 2 Moredun Research Institute, Bush Loan, Scotland, Penicuik EH26 0PZ UK

**Keywords:** Ovine pulmonary adenocarcinoma, OPA, Jaagsiekte, Ultrasonography, Disease control

## Abstract

**Background:**

This ultrasonographic study monitored lesions involving the lung surface suspected to be the early stages of ovine pulmonary adenocarcinoma (OPA) tumours over 4 months in commercially farmed sheep. The enlargement of these lesions defined ultrasonographically, which likely represent the development of OPA tumours, have important implications for ultrasound screening schedules in veterinary management plans attempting to eliminate OPA by test-and-cull.

**Results:**

The lungs of 58 adult Scottish Blackface sheep with ultrasonographic changes at the lung surface consistent with early OPA tumours were examined two to six times over 40 to 290 days. Lesion development, represented in early video recordings by 2–3 mm lesions involving the visceral pleural and comet tails, then a decreasing length of the hyperechoic line representing the normal visceral pleura and increasing depth of the sharply-demarcated and largely uniform hypoechoic areas into the lung parenchyma, was found in 26 of the 58 sheep. The rate at which the sonographic lesions progressed varied considerably and in 10 of 17 Group 1 sheep developed quickly from an estimated depth of 2–30 mm up to 70 mm between 60 and 120 days later. These sonographic lesions were confirmed as OPA at necropsy; histological changes of concurrent bacterial infection were detected in one of these 10 Group 1 sheep. Thirty-one sheep had sonographic changes ≤30 mm consistent with very early OPA at the first examination which had reduced or were not observed at subsequent examination. Five of these 31 sheep were necropsied, 3 had small OPA lesions while 2 had no significant pathology.

**Conclusion:**

Lesions involving the visceral pleura, with sonographic changes consistent with previous published findings of early OPA, developed over 40–120 days to large masses in 10 of 17 Group 1 sheep with the provisional sonographic diagnosis confirmed histologically at necropsy. While it is possible that atalectic lung could have caused some of the minor sonographic changes there was no microscopic evidence of pathologies other than OPA in nine of 10 Group 1 sheep. We conclude that some small tumours progress to large tumours within 3 months questioning the assumption that OPA is a slow growing tumour in adult sheep taking several years to cause clinical disease. The findings that a proportion of small ultrasonographic lesions are not found again at subsequent scanning illustrates the challenges of interpreting small (< 1–2 cm) lesions during rapid whole flock ultrasonographic examination and we continue to recommend re-scanning suspicious sonographic changes 2 months later.

**Electronic supplementary material:**

The online version of this article (10.1186/s13620-018-0134-0) contains supplementary material, which is available to authorized users.

## Background

Ovine pulmonary adenocarcinoma (OPA) is an infectious lung cancer of sheep. OPA is relatively common in Ireland, where it is on the DAFM list of notifiable diseases (Part B), although less common here than in Northern Ireland or the UK. Veterinary investigation reports for submissions in the 5 years 2012–2016 show that in Northern Ireland there were on average 21 diagnoses of OPA per year representing up to 35% of respiratory disease diagnoses and > 3% of all disease diagnoses; in the U.K. there was an average of 76 OPA diagnoses per year representing 20% of respiratory disease diagnoses and 1% of total sheep diagnoses; whilst in Ireland OPA was less commonly diagnosed at < 1% of respiratory diagnoses [1, 2]. Passive surveillance data represent a gross underestimation of disease prevalence and this is highlighted by the identification of more than 500 OPA-affected sheep by transthoracic ultrasonography of almost 37,000 adult sheep in 2016 [3]. Recent abattoir surveys in Ireland and the UK, found OPA in 0.6% and 0.9% respectively of lungs from adult sheep [4, 5]. This too is likely to be an underestimate as it does not account for sheep dying on farm, which from a UK study was 6% of fallen stock [6]. Whatever the true figure, it is clear that OPA is causing significant losses to the sheep industry and that controlling the spread of this disease is currently an unmet need. Furthermore, the welfare of OPA-affected sheep with moderate to advanced clinical disease must not be underestimated.

OPA is caused by Jaagsiekte sheep retrovirus (JSRV) which is transmitted mainly via the respiratory route but also by milk or colostrum [7, 8]. The virus infects and transforms type II alveolar epithelial cells and Clara cells [9] leading to the development of tumours that can occupy a large proportion of the lungs. The tumour cells produce JSRV [10]. In some sheep copious fluid, produced by the OPA-affected lung, emerges via the nostrils. This fluid contains large amounts of JSRV [11] but even without such noticeable lung fluid JSRV produced by the tumour cells can infect other sheep in the same airspace [12]. Once the clinical signs such as loss of body condition, increased respiratory effort and increased lung secretions (positive “wheelbarrow test”) appear, the disease is already at an advanced stage and is invariably fatal [13]. The ability to detect JSRV in samples of blood, milk or colostrum is greater in sheep with clinical OPA compared to pre-clinical animals [8, 14] indicating that sheep with advanced OPA tumours pose the greatest risk for JSRV transmission.

As there is still no commercially-available effective diagnostic test for early OPA or JSRV infection and no treatment nor vaccine [15, 16] we have re-visited the use of trans-thoracic ultrasonography to identify and cull pre-clinical OPA cases [17]. Ultrasound examination will not detect all tumours < 1–2 cm. Nevertheless, culling sheep with > 2–3 cm OPA lesions from a flock as early as possible should reduce JSRV transmission within the flock and recent field trials have yielded encouraging preliminary results [18]. The optimal time interval for re-screening sheep will depend upon the rate at which tumours develop from undetectable size to > 2–3 cm when a specific diagnosis is much more likely [19]. However, little is known about the rate of tumour growth in OPA-affected sheep and there are no previous reports regarding the rate of OPA development in sheep managed commercially where concurrent endemic diseases, periods of poor nutrition, and metabolic stress may exert influences not observed in sheep kept under controlled management conditions.

Experimental studies have demonstrated that younger sheep are more susceptible to JSRV infection and that the time taken and probability for infection to develop into OPA lesions is inversely correlated with age [20]. In experimental infections, OPA in young lambs can rapidly advance to clinical disease in 7–9 weeks [21]. However, naturally-occurring OPA is most commonly diagnosed in 2 to 4 year-old sheep [13]. It has been assumed, though never proven, that most naturally-occurring OPA tumours grow slowly from an early age, possibly over several years, and OPA has often been referred to as a “slow disease” [22, 23]. Caporale and others [24] reported detection of JSRV infection in peripheral blood samples with development of clinical OPA 27 months later but did not assess the rate of tumour development. We previously described a ram kept in a veterinary hospital facility where an OPA lesion of approximately 2–3 cm (as determined by ultrasound scan) grew to approximately 8 cm by 15 cm over 30 months with only mild clinical signs [19]. Another study monitored 16 sheep naturally affected by OPA using computed tomography every 3 months over 2 years and showed that lesions progressed slowly in the majority of sheep [25].

The study described here was undertaken in sheep managed under commercial farming conditions to serially record ultrasound scans of lung lesions involving the visceral pleura consistent with published reports of OPA from the earliest detectable stages and to conduct detailed gross and histological examination at necropsy. The confirmation of OPA, and the absence of other pathological processes within the lung parenchyma, would suggest that the increase in the sonographic abnormalities over the time series represented the development of naturally-occurring OPA tumours. These data will help guide disease control and possible OPA elimination from infected flocks, as well as informing the design of potential accreditation schemes and any future prevalence studies.

## Methods

The aim of this study was to characterise the development of suspected early OPA lesions identified by ultrasonography in naturally affected sheep managed under commercial farming conditions. During August to September 2017, all 7820 adult Scottish Blackface sheep on four hill farms were examined by trans-thoracic ultrasonography using a 6.5 MHz micro-array probe connected to a real-time B-mode ultrasound machine (DP50; BCF Technology) as part of a 3 year on-farm screening programme testing the hypothesis that it is possible to reduce/eliminate OPA from known infected flocks. Sheep were examined as described previously [19, 26], at a rate of up to 120 per hour. An additional 1–2 min was taken to further investigate suspected OPA cases and record the sonographic changes using video capture software (Elgato, www.elgato.com). The depth of the largest sonographic change was measured in a horizontal plane from the visceral pleura to the most distal margin of the sharply-defined hypoechoic area on the video recordings. This measurement provided an estimate of lesion depth and enabled assessment of lesion development in a non-invasive manner. In addition, development of lesions at the lung surface could be visualised in the serial video recordings as replacement of the hyperechoic line, representing the visceral pleura, by the largely uniform hypoechoic and well-defined area giving such lesions a triangular shape during the early stages of development.

Sheep found to have a sharply-demarcated, triangular-shaped, and uniformly hypoechoic area at the ventral lung margin extending > 30 mm from the visceral pleura, consistent with a sonographic diagnosis of OPA [17, 19, 26–28], were immediately culled from the flock. Sheep which showed sonographic lesions, including numerous comet tails originating from 1 to 3 mm anechoic circles over a large area of the visceral pleura of one or both lungs and/or single 5–30 mm sharply-demarcated hypoechoic triangular-shaped areas consistent with lung consolidation were noted and 17 of these sheep were brought together from 4 farms to form Group 1. These sheep were re-scanned on five occasions over the following 16 weeks. All sheep were rapid scanned by the same person (PS) without reference to tag number or previous results using a routine developed to scan the ventral lung lobes cranially to caudally at two levels referenced to the sheep’s ipsilateral elbow. The sheep were euthanised when clinical signs of respiratory disease became apparent during gathering, when the tumours extended more than 60 mm from the visceral pleura or at the end of the 4 months’ study. The lungs were removed for post mortem examination (PME), photographed and samples taken for histopathology as previously described previously [19]. Some of the remaining sheep with small lesions suspicious of OPA stayed on their respective farms (Group 2) managed separately from the main flocks, and 42 were re-scanned later, the timings of which were determined by flock management. These sheep were not available for necropsy. Other sheep were culled from the flocks and were not available for re-scanning.

## Results

Of 7820 sheep scanned on the 4 farms 104 had lesions indicative of OPA larger than 30 mm and were culled. Seventy nine had small sonographic changes suspected to be OPA and 17 of these became animals group 1 pastured together. No Group 1 sheep had abnormal respiratory signs and all were in very good to excellent body condition at the start of the study (Body condition score > 3.5; scale 1–5) consistent with the absence of clinically significant chronic disease, but some lost condition during the course of the study (Table 1).

**Table 1:** Results of ultrasonography and PME of sheep in group 1 (sheep pastured together and re-scanned at regular intervals)

Sheep	lung	Approx. depth of hyperechoic area (mm)						Post mortem results				
ID		Weeks from first scan						visible	LF	BCS	OPA	Other
No.		0	6	9	11	13	16	OPA	(ml)	(0–5)	histo	path
1337	L	10	15	30	40	35	30	Early	0	3.5	Pos	
	R	0	0	0	0	0	0					
5381	L	0	0	3	0	3	10	Adv	4	3.5	Pos	pl
	R	20	30	30	30	40	40					
18823	L	30	35	40	50	50	50	Mod	0	3	Pos	
	R	0	0	0	0	0	0					
15786	L	5	40	50	60	60	70	Adv	6	3	Pos	pl
	R	0	0	0	0	8	20					
2806	L	15	20	20	30	25	40	Pos?	0	4	Pos	
	R	10	0	10	10	10	20					
4777	L	3	3	15	30	40		Adv	0	2	Pos	
	R	3	50	50	60	60						
15733	L	20	30	40	50	60		Adv	20	3	Pos	pl
	R	0	3	30	40	40						
5466	L	15	25	50	40			Adv	40	2	Pos	
	R	50	50	60	60							
4709	L	20	40					Adv	10	3.5	Pos	pl
	R	2	40									
2584	L	0	0					Mod	0	3	Pos	b
	R	2	30									
4803	L	0	0	0	0	0	0	Neg?	0	3.5	Pos	b,p
	R	25	3	0	2	2	5					
5182	L	0	0	0	0	0	0	Neg?	0	3.5	Pos	p
	R	10	20	0	0	0	0					
6743	L	15	0	0	0	0	0	Neg	0	4	Neg	p
6750	L	2	0	0	0	3	0	Neg?	0	3	Pos	
11641	L	15	0	0	0	0	0	Neg?	0	3	Neg	p
16610	L	3	5	25				Neg	ND	0.5	Neg	b
	R	30	60	60								

The approximate depth of the hypoechoic area was determined from videos of the sonograms. Visible lesions indicative of OPA at PME were classified as follows: affecting > 40% of the lung (Adv), affecting 10–40% of the lung (Mod), affecting < 10% of the lung (Early), > 3 consolidated lesions (Pos?), 1–3 discrete consolidated lesions < 1 cm (Neg?), No visible lesions (Neg). LF: Volume of lung fluid collected by tipping after euthanasia. BCS: body condition score (scale 1–5). OPA histo: histological confirmation of OPA status. Other path: signs of bacterial (b) or parasitic (p) pathologies by gross PME and/or histopathology, pleurisy (pl)

**Fig. 1:**
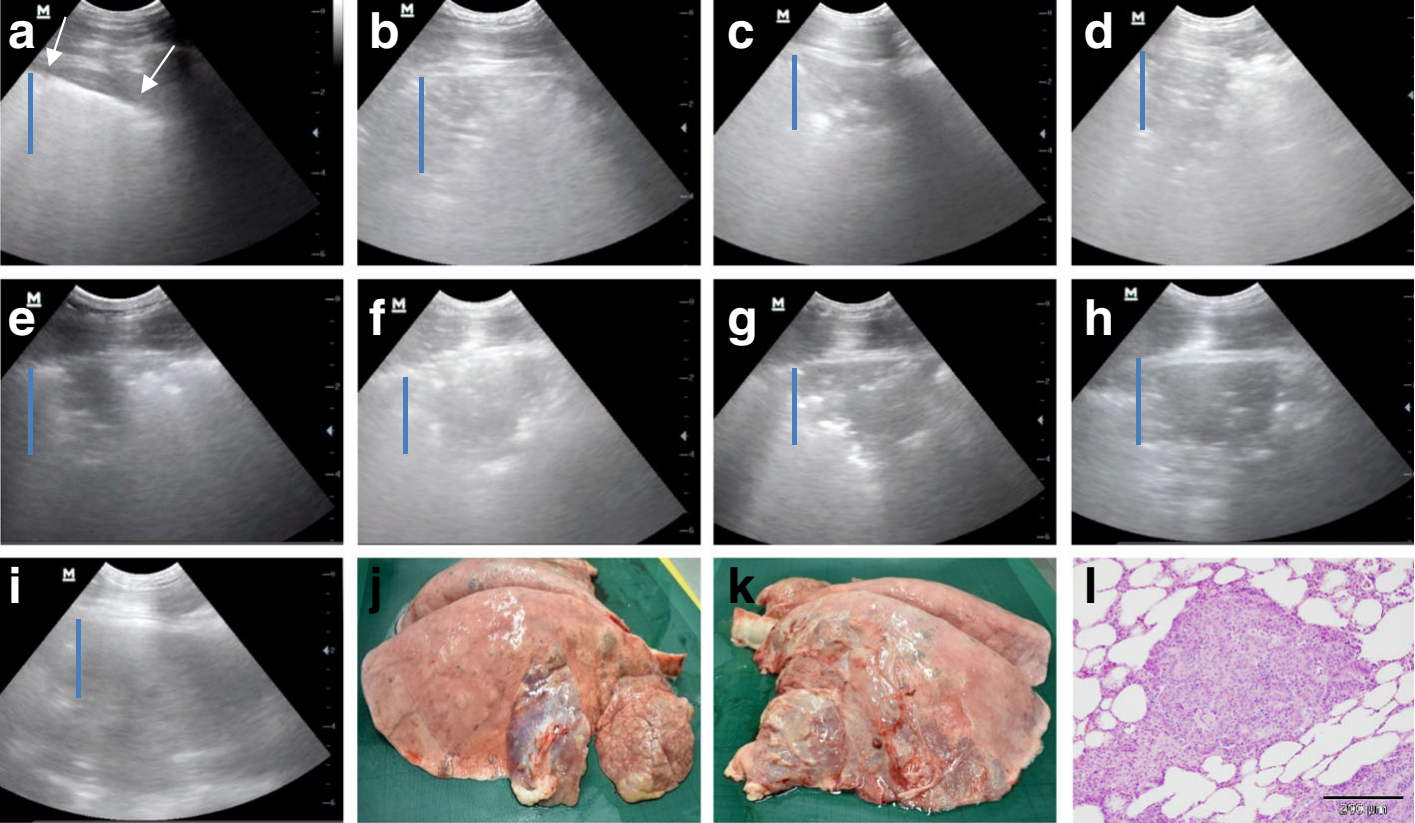
**a**–**i** Ultrasound recording of the chest of sheep 15733 a-d: right side, **e**–**i** left side. Dorsal is to the left of the image. Scale (cm) is on the right margin. The blue bar represents 2 cm. At day 0 no ultrasonographic abnormality was detected in the right lung. **a** Week 6: The hyperechoic line representing the visceral pleura (normal lung surface) is clearly visible and contains two 2 mm hypoechoic circles (arrows). **b** Week 9: A poorly-defined hypoechoic triangular area extends about 20 mm from the visceral pleura in the ventral lung field. **c** Week 11: hypoechoic area extends about 20 mm. **d** Week 13: hypoechoic area extends about 30 mm. **e** Day 0: A poorly-defined, hypoechoic triangular area extends about 20 mm from the visceral pleura. Several 2–5 mm hypoechoic circular areas occur immediately ventral to the triangular lesion. **f** Week 6: The hypoechoic triangular area extends about 30 mm and is considerably wider than on day 0 such that no normal lung surface is visible. **g** Week 9: The hypoechoic triangular area extends about 30 mm and its base fills the field of view. **h** Week 11: The hypoechoic triangular area extends about 45 mm and its base fills the field of view. **i** Week 13: The hypoechoic area extends more than 50 mm from visceral pleura and fills the field of view. **j** Necropsy at week 13 showed OPA affecting the middle lobe of the right lung. **k** Consolidation affecting a large proportion of the ventral aspects of the left lung lobes. Fibrous pleurisy is also present, indicative of prior inflammation and suggesting a secondary bacterial infection. **l** Photomicrograph of haemotoxylin and eosin stained lung section from sheep 15733 showing typical lesions of OPA


Sixteen video recordings (Additional file 1: Video S1, Additional file 2: Video S2, Additional file 3: Video S3, Additional file 4: Video S4, Additional file 5: Video S5, Additional file 6: Video S6, Additional file 7: Video S7, Additional file 8: Video S8, Additional file 9: Video S9, Additional file 10: Video S10, Additional file 11: Video S11, Additional file 12: Video S12, Additional file 13: Video S13, Additional file 14: Video S14, Additional file 15: Video S15, Additional file 16: Video S16, Additional file 17: Video S17) detailing development of the ultrasonographic changes in two sheep from Group 1 and one sheep from group 2, with OPA being the only respiratory disease confirmed at necropsy, are available and a series of sonograms screen-grabbed from these recordings for one sheep are described in Fig. 1. The ultrasound recordings were made without skin preparation at the recording sites on the chest wall and there has been no digital enhancement of recordings. These recordings therefore represent the images veterinary practitioners will generate under farm conditions.

Additional file 1:15733 Right Lung Video A (week 6). Week 6: The hyperechoic line representing the visceral pleura (normal lung surface) is clearly visible and contains several very small lesions on the visceral pleura and associated comet tails. No ultrasonographic abnormalities were recorded in this lung at the first recording and the sheep was selected for Group 1 on the basis of the sonographic changes observed in Additional file 5: Video S5. (MP4 6229 kb)



Additional file 2:15733 Right Lung Video B (week 9). A poorly-defined hypoechoic triangular area extends about 20 mm from the visceral pleura in the ventral lung field. The heart is imaged to the right (ventrally) at the start of the recording and the diaphragm towards the end of the recording appears as a bright convex hyperechoic band. (MP4 7971 kb)



Additional file 3:15733 Right Lung Video C (week 11). A poorly-defined hypoechoic area extends about 20–30 mm from the visceral pleura in the ventral lung field and occupies the whole field. There are numerous comet tails dorsal to this lesion. (MP4 11345 kb)



Additional file: 415733 Right Lung Video D (week 13). A poorly-defined hypoechoic area extends about 30 mm from the visceral pleura in the ventral lung field immediately dorsal to the heart base. During some of the recording the lesion extends to occupy the whole field. (MP4 8483 kb)



Additional file 5:15733 Left Lung Video E (day 0). The hyperechoic line formed by the normal lung surface is clearly visible at the start of the video recording. A triangular hypoechoic area is seen extending for about 20 mm from the lung surface immediately dorsal to the heart base. There are several comet tails arising from the visceral pleura dorsal to the lesion. Dorsal to the left of the recording. (MP4 4902 kb)



Additional file 6:15733 Left Lung Video F (week 6). A triangular hypoechoic area extending 30 mm from the visceral pleura is seen immediately dorsal to the heart base. There are several 2–3 mm anechoic circles on the visceral pleural with comet tails distally which appear separate to the main lesion although it is not possible to be certain that therse lesions are not confluent. (MP4 9832 kb)



Additional file 7:15733 Left Lung Video G (Week 9). The hypoechoic triangular area extends about 30 mm and its base fills the field of view. There are several 2–3 mm anechoic circles on the visceral pleural with associated comet tails immediately dorsal to the lesion. The heart chambers are clearly imaged with the heart base displaced off the chest wall by the lesion. (MP4 15208 kb)



Additional file 8:15733 Left Lung Video H (Week 11). The hypoechoic triangular area immediately dorsal to the heart base extends about 45 mm and its base fills the field of view. The heart chambers are clearly imaged with the heart base displaced off the chest wall by the lesion. (MP4 8714 kb)



Additional file 9:PP15733 Left Lung Video I (Week 13). The hypoechoic area extends more than 50 mm from visceral pleura and fills the field of view. The lesion has a “hepatoid” appearance. The diaphragm is imaged ventrally towards the end of the recording appearing as a broad convex hyperechoic band. (MP4 7036 kb)



Additional file 10:15786 Video J (Week 0). Left lung imaged first with 15 s interval as the sheep is turned then right lung is scanned. There are several 5–8 mm hypoechoic lesions involving the visceral pleura at the ventral margin of the left lung. The heart chambers are clearly visible. There are several comet tails arising from the visceral pleura. (MP4 15282 kb)



Additional file 11:15786 Video K (Week 6). A sharply-defined hypoechoic triangular area immediately dorsal to the heart base extends about 40 mm from the visceral pleura and its base fills the field of view. The right lung is not imaged. (MP4 6629 kb)



Additional file 12:15786 Video L (Week 9). The sharply-demarcated hypoechoic mass in the ventral left lung extending for about 50 mm has displaced the heart off the chest wall. The right lung is normal represented by the continuous hyperechoic line which represents the lung surface. (MP4 9829 kb)



Additional file 13:15786 Video M (Week 11). The sharply-demarcated hypoechoic mass in the ventral left lung extending for about 60 mm has displaced the heart off the chest wall. There are several comet tails arising from the surface of the right lung surface. (MP4 17160 kb)



Additional file 14:15786 Video N (Week 13). Note that the right lung is scanned first, then the left lung in this recording. There are several 3–5 mm anechoic circles involving the surface of the right lung surface with associated broad comet tails. The sharply-demarcated hypoechoic mass in the ventral left lung extending for about 60 mm has displaced the heart off the chest wall. (MP4 14172 kb)



Additional file 15:15786 Video O (Week 16). Note that the right lung is scanned first, then the left lung in this recording. The sharply-demarcated hypoechoic mass in the ventral left lung extends for more than 60 mm and has displaced the heart off the chest wall. There are numerous comet tails arising from the visceral pleural dorsal to this lesion. (MP4 27802 kb)



Additional file 16:5217 Video P (Week O). There are numerous comet tails, associated with very small (1–2 mm) anechoic circles, arising from the visceral pleura of both lungs. (MOV 11354 kb)



Additional file 17:5217 Video Q (Week 16). The sharply-demarcated hypoechoic mass in the ventral left lung extends for about 60 mm and has displaced the heart off the chest wall. There are several 3–5 mm hypoechoic areas involving the visceral pleura dorsal to the main lesion. There is 15 mm deep sharply-demarcated triangular lesion at the ventral margin of the right lung which displaces the base of the heart from the chest wall. There are numerous very small anechoic lesions involving the visceral pleura from which originate comet tails. (MP4 17044 kb)


Development of the sonographic changes was highly variable in those sheep subsequently confirmed as OPA at necropsy whereby some lesions increased rapidly, others at a much slower rate, and some reduced in size (Table 1). The lesion growth rate, defined as the depth of the largest observed hypoechoic area from the visceral pleura (see Additional files 1, 2, 3, 4, 5, 6, 7, 8, 9, 10, 11, 12, 13, 14 and 15 for two Group 1 sheep), ranged from − 0.08 to + 0.95 mm/day (mean 0.3 mm/day). For 10 of the 17 sheep in Group 1, the size of the sharply-demarcated hypoechoic area increased during the course of the study and OPA was confirmed by histology; there were no histological findings of other disease processes in the lungs except for sheep 2584 which had lesions consistent with a secondary bacterial infection. All 10 sheep were in good body condition when culled and none showed respiratory distress at rest. Five sheep had sonographic changes consistent with OPA in one lung only and macroscopic lesions of OPA were confirmed in that lung at necropsy. Four sheep had several millimetres of fibrous pleurisy covering the visceral surface of the OPA tumours but this rarely extending onto normal lung (Fig. 1).

Several ultrasonographic changes considered suggestive of early OPA were not visible at subsequent examinations (Table 1). Four sheep in group 1 had small sonographic lesions at the first scan but no discernible lesions at subsequent examinations. Gross PME showed that two of these sheep were clear of OPA and two had small lesions of OPA in a position coinciding with the ultrasonographic findings of the first scan and these findings were confirmed by histology. One sheep (4803) had a lesion which appeared to reduce in size over the course of the study which was probably the abscess visible grossly at PME, but 2 small OPA lesions were also found and confirmed by histology.

One sheep (16610) had a lesion that increased in depth but when the sheep died without premonitory signs there was histological evidence of bacterial infection only. Sheep 2584 also died unexpectedly during the monitoring period; severe suppurative pneumonia as well as OPA were noted at PME and confirmed by histology. The third sheep that died unexpectedly was unavailable for necropsy and is not included in Table 1.

Group 2 comprised 42 sheep with sonographic findings not inconsistent with very early OPA (including comet tails and small 1–3 mm anechoic circles involving the visceral pleura and/or single sharply-demarcated 5–15 mm hypoechoic triangular-shaped areas) that were classified as “inconclusive” remained on the farms and were re-scanned 3–6 months later. The results (Table 2) add weight to the results for group 1 in that development of suspected OPA lesions identified by ultrasonography varied from very rapid growth to reduction or disappearance; 16 had ultrasonographic lesions that had increased in size at subsequent scans. These sheep were not available for PME but the sonographic appearance was similar to sheep with confirmed OPA in Group 1 and our previous work [17, 19, 26–28], and therefore we can presume that the majority were indeed OPA. Twenty-six had no sonographic lesion when re-scanned.

**Table 2:** Results of ultrasonography of sheep in group 2 (sheep remaining on their farms of origin and re-scanned at various intervals). The approximate depth of the hypoechoic area (mm) was estimated from videos of the ultrasonographs. Results are shown only for the side of the lungs with the more advanced lesions

Sheep	Time from first scan (weeks)						
ID No.	0	8	12	16	24	30	40
13011	2				2	40	60
6092	30					60	
6217	5			5	60		
13186	2				50		
15976	2	5		60			
5217	3			60			
10445	20			60			
2262	5			30			
4805	30			50			
11171	20		30	30			
7503	10		60				
2799	30	60					
6371	20	40					
8080	3	30					
4143	2	20					
6432	3	20					

In addition, 26 sheep had lesions ≤30 mm at the first scan and no lesion was detected at the subsequent scan 8–40 weeks later

## Discussion

Our recent work has shown the value of veterinary ultrasonographic examination of suspected cases of OPA [19] supporting earlier work describing characteristic sonographic changes in this and other ovine respiratory diseases [17]. This study presents some of the additional data necessary to optimise the design of a veterinary practitioner-led transthoracic ultrasound scanning protocol that is an affordable pre-clinical diagnostic test suitable for either OPA surveillance or disease control applications, both of which have previously been hampered by the lack of a reliable ante-mortem diagnostic test. Experience acquired with ultrasonographic examination over several months [3, 26–28] enabled up to120 adult sheep to be examined per hour. The video recordings included in this report were taken on-farm and represent the sonographic changes veterinary practitioners will observe when using similar ultrasound equipment which is widely available in veterinary practice. Rapid ultrasound examination undertaken by the veterinary practitioner proves cost neutral to a farmer whose flock has an OPA prevalence above 1.5–2% [26]. Since in an abattoir survey in Ireland 1.6% of lungs were positive for JSRV [4] many OPA-affected flocks in Ireland will have an OPA prevalence above 1.5% and should see an immediate financial gain from whole flock screening. These costings do not take into account any future benefits accrued from reducing OPA prevalence or successful treatment of sheep identified with bacterial infections such as fibrinous pleurisy [28]. Furthermore, handling of all adult sheep and assessing body condition scores will determine whether there are any other concerns allowing immediate veterinary intervention. The animal welfare benefits of prompt culling of sheep before extensive lung and pleural pathologies develop must also be taken into account.

Our results highlight a number of important findings pertinent to the implementation and optimisation of OPA screening by ultrasonography. Firstly, that sonographic findings of several 1–3 mm anechoic circles at the visceral pleura and associated comet tails, then well-demarcated 10–20 mm hypoechoic areas involving the lung surface, developed into large 50–70 mm OPA tumours within 3–4 months. However, it should be noted that the lesion that develops is not a single clonal tumour expanding but is more likely to be a coalescence of multiple tumour initiation events arising as a consequence of JSRV infection of its target cells (type II pneumocytes in this anatomical location). Previous work has indicated the multiclonal nature of OPA tumours [29] [30]. It is possible that several/multiple OPA lesions within the lung parenchyma, which would not have been detected during the ultrasound examination because aerated lung separated them from the lung surface, could have coalesced to contribute to the apparent rapid increase in depth of the original superficial hypoechoic area(s). However, no macroscopic lesions suggestive of OPA were observed in lung cross sections at necropsy which did not also involve nodules in contact with the visceral pleura. While CT studies [25] showed lesions within the lung parenchyma of an advanced OPA case, similar lesions were also present involving the overlying visceral pleura which would have been detected ultrasonographically. Secondly, irrespective of the origin of the OPA lesions detected during ultrasound examination, without pre-clinical detection, sheep with OPA may be present in the flock shedding virus and potentially infecting flock-mates for several months at least. As OPA lesions appear to develop to large masses well within the 6 to 12 months ultrasound scanning intervals presently recommended this suggests shorter time intervals between flock screens would be desirable in order to cull sheep with pre-clinical OPA as early as possible. However, such short scanning intervals need to be balanced against increasing veterinary costs and providing access to sheep during the annual production cycle of extensively-managed flocks. The factors controlling the rate of OPA development are not yet known but are likely to be multi-factorial including concurrent respiratory infections, host genetics, immune responses, and metabolic stress.

Our study again highlights the problem of interpreting very small ultrasonographic lesions at a single examination whereby 4 sheep in group 1 with initial sonographic changes showed no evidence of OPA when they were re-scanned 1–3 months later but 2 had evidence of small OPA lesions at necropsy, illustrating that small lesions can easily be missed. Furthermore, 2 had no evidence of OPA suggesting the initial sonographic changes may have been due to some other lesion such as small abscesses or worm cysts, although regression of OPA lesions cannot be excluded [25].

These field studies are still in progress and the sheep will continue to be monitored. Such a strict interpretation of ultrasound changes may be considered overzealous but it could be reasoned that, where examinations extend to 6–12 months intervals, biocontainment of those sheep with suspicious lesions is essential otherwise significant OPA lesions could develop during these time periods presenting a major risk for JSRV transmission within the flock. Due to geographical location, many of the flocks in the present study involve a 3 to 4 h return journey for the authors restricting the opportunity for re-examination of small numbers of sheep with suspicious ultrasonographic findings. Veterinary surgeons dealing with their own clients could include such re-examinations into their regular farm visits allowing more timely interventions; an interval of around 2 months is recommended based upon the results presented here.

Another finding from this study relevant to on-farm rapid scanning is that many sheep had gross lesions of OPA correctly detected by ultrasound in one lung only, highlighting the need to examine both sides of the chest. All 10 sheep in Group 1 with large OPA lesions were in good to excellent body condition when culled and none showed respiratory distress at rest. This shows that culling sheep on the basis of poor condition, which is commonly practised by farmers in an attempt to reduce OPA prevalence, will not remove pre-clinical OPA-affected sheep and there are many other endemic diseases which cause significant weight loss in adult sheep. The high prevalence of fibrous pleurisy in OPA-affected sheep highlights the association between OPA and previous bacterial lung infections but does not indicate causation.

## Conclusion

The use of ultrasound scanning over several months has, for the first time in sheep managed under commercial farming conditions, enabled us to follow the development of small superficial lung lesions, shown at necropsy to be OPA tumour. The increase in size of these sonographic changes may represent rapid development of naturally-occurring OPA tumours in some adult sheep and slower development of early OPA lesions in others. The coalescence of OPA tumours at the visceral pleura with tumour nodules developing within the lung parenchyma, undetected by ultrasound examination, may contribute to the apparent rapid OPA lesion development observed in this study. Whether OPA develops from a single clonal tumour or, more likely, by coalescence of multiple tumours, our data indicate that in some sheep there is rapid development of lesions defined ultrasonographically. This contradicts the dogma that OPA is a slow growing tumour that starts in young lambs and takes 2–4 years to produce clinical disease shown at necropsy to be OPA.

The potential for rapid tumour growth, irrespective of whether predominantly at the lung surface, within the lung parenchyma or both lung areas, resulting in production of large amounts of JSRV in some sheep must be taken into account when developing a disease control plan in OPA-affected flocks or when designing a flock accreditation scheme. This study supports our current recommendation that, where small sonographic lesions potentially indicative of OPA are found, sheep should be quarantined and re-examined around 2 months later.

Results from the present study have important implications for the design of protocols aimed at reducing OPA incidence by ultrasound screening and for the discussions that veterinary practitioners must have with clients when advising on ultrasound scanning interval including the limitations of this diagnostic procedure. Similarly, the dynamics of tumour growth must be considered if ultrasound screening is to be used as the basis of a flock accreditation scheme or to estimate OPA prevalence in the national flock.
